# Divergent immune cascades activated by metabolic versus mechanical exercise intensity: revisiting cytokine heterogeneity in “moderate-intensity exercise”

**DOI:** 10.3389/fimmu.2026.1846052

**Published:** 2026-05-13

**Authors:** Jia-wen Wang, Jian-Jun Xun, Li-Na Jian

**Affiliations:** 1 Department of Orthopedics, The Fourth Hospital of Hebei Medical University, Shijiazhuang, Hebei, China; 2 Department of Pain Rehabilitation, The Fourth Hospital of Hebei Medical University, Shijiazhuang, Hebei, China

**Keywords:** cytokine heterogeneity, dual-axis classification, exercise immunology, IL-6 signaling, macrophage polarization, meta-analytic heterogeneity, myokine, temporal inflammatory profiling

## Abstract

“Moderate-intensity exercise” is widely regarded as anti-inflammatory; however, meta-analyses of key cytokines have yielded highly inconsistent results. We argue that this inconsistency reflects not mere statistical noise, but a fundamental conceptual conflation: aerobic exercise intensity is anchored to metabolic rate (%VO_2_max), whereas resistance training intensity is anchored to neuromuscular force output (%1RM). These quantify orthogonal dimensions of physiological stress and are not physiologically interchangeable, yet the shared label “moderate intensity” groups them into a single exposure category. Crucially, these two exercise modalities activate inflammatory signaling pathways in opposite directions: aerobic exercise engages a myokine-mediated anti-inflammatory axis (IL-6→AMPK→IL-10), whereas resistance exercise initiates a damage–repair immune cascade (DAMPs→M1→M2 macrophage polarization), yielding fundamentally divergent acute cytokine profiles. The aggregation of incommensurable physiological stimuli is a major contributor to the extreme heterogeneity reported in current meta-analyses. The proposed “dual-axis classification” quantifies metabolic and mechanical dimensions in parallel rather than as mutually exclusive categories; for mixed-modality interventions (e.g., HIIT), both dimensions should be reported concurrently. Accordingly, we recommend that future studies adopt a dual-axis framework that reports metabolic and mechanical loads separately, implement time-resolved inflammatory sampling, and apply causal inference methods to re-stratify existing evidence by exercise modality, thereby addressing this central source of inconsistency.

## 1 Introduction

Interleukin-6 (IL-6), one of the most pleiotropic cytokines in immunity, exerts either pro- or anti-inflammatory downstream effects depending primarily on the nature of the upstream stimulus and the mode of signal transduction (1, 2). Specifically, the membrane-bound receptor–mediated classic signaling pathway predominantly confers anti-inflammatory and tissue-protective effects, whereas the soluble receptor–mediated trans-signaling pathway tends to drive pro-inflammatory and pathological responses (2, 3). This dichotomy indicates that increases in circulating IL-6 cannot be interpreted uniformly; even at comparable concentrations, IL-6 may reflect an anti-inflammatory myokine pulse released from contracting skeletal muscle (4), or, alternatively, a pro-inflammatory cascade driven by tissue damage or chronic inflammation (1).

However, current meta-analyses in exercise immunology often overlook this distinction, pooling IL-6 responses from heterogeneous exercise modalities—such as aerobic exercise and resistance training—into a composite “exercise” intervention group, without stratification by upstream stimulus type (metabolic vs. mechanical) (5, 6).

The issue is not primarily the quality of individual studies, but rather that aerobic and resistance exercise are placed within a shared intensity framework. A 2025 joint expert statement by the American College of Sports Medicine and the Exercise and Sports Science Australia clarifies that aerobic exercise intensity is anchored to the percentage of maximal oxygen uptake (%VO_2_max) or metabolic equivalents (METs), indexing whole-body metabolic rate, whereas resistance training intensity is anchored to the percentage of one-repetition maximum (%1RM), indexing neuromuscular force output (7). These two systems quantify orthogonal dimensions of physiological stress and are not physiologically convertible; yet, in both research and guidelines, they are subsumed under shared qualitative descriptors such as “moderate” or “vigorous” (7). As a result, aerobic and resistance training are treated as the same exposure in meta-analyses.

Consistent with this concern, meta-analyses report substantial between-study heterogeneity for inflammatory markers following exercise interventions, with I² values of 72% for C-reactive protein (CRP), 97% for IL-6, and 91% for tumor necrosis factor-α (TNF-α) (8). Subgroup analyses stratified by exercise modality provide further support: aerobic training demonstrates relatively consistent reductions in IL-6 and CRP, whereas resistance training yields more heterogeneous and often non-significant effects on IL-6 and TNF-α (9–11).

**Figure 1:**
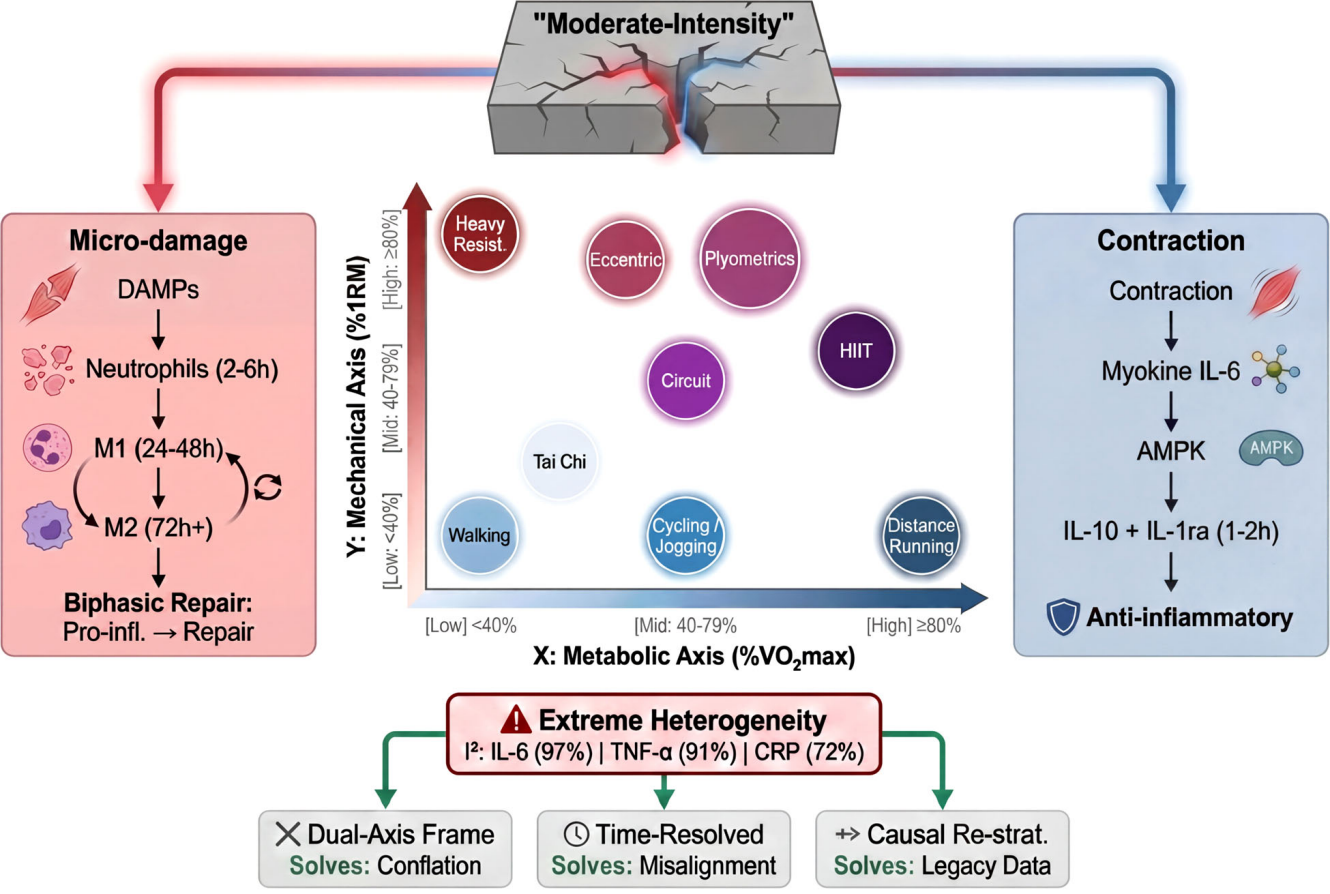
The metabolic–mechanical coordinate plane: reframing “moderate-intensity exercise” as a two-dimensional exposure. The figure replaces the conventional one-dimensional “moderate-intensity” label with a two-dimensional coordinate system in which any exercise bout occupies a unique position defined by its metabolic load (x-axis, anchored to %VO_2_max, blue gradient) and mechanical load (y-axis, anchored to %1RM, red gradient); both axes are partitioned into Low (<40%), Mid (40–79%), and High (≥80%) strata. Color saturation encodes magnitude on each axis, while hue indicates which axis predominates: predominantly blue points represent metabolically dominant stimuli (walking, cycling/jogging, distance running), with Tai Chi positioned near the origin as a low-load reference exemplar; predominantly red points represent mechanically dominant stimuli (heavy resistance training); and purple points represent mixed-modality interventions loading both axes substantially (HIIT, circuit resistance training, plyometrics, eccentric exercise). The fractured block at the top denotes that the operational label “moderate-intensity exercise” obscures this two-dimensional structure when used as an unstratified pooling criterion in evidence synthesis; the red and blue light streams emerging from the fractures channel into the two flanking mechanism boxes, illustrating the decomposition of the unified label into its two divergent biological axes. The left red box summarizes the mechanical axis cascade: microstructural damage → DAMPs → neutrophil infiltration (2–6 h) → M1 macrophage polarization (24–48 h) → M2-mediated repair (72 h+), yielding a biphasic pro-inflammatory-to-resolution profile. The right blue box summarizes the metabolic axis cascade: sustained contraction → myokine IL-6 → AMPK → IL-10 + IL-1ra (peak 1–2 h) → systemic anti-inflammatory effect. The lower section illustrates the consequence and proposed remedies. Pooling exposures across this 2-D plane without stratification produces extreme heterogeneity in cytokine meta-analyses (I²: IL-6 = 97%, TNF-α = 91%, CRP = 72%). Three recommendations are mapped explicitly to the heterogeneity sources they address: (i) dual-axis classification resolves axis-level conflation; (ii) time-resolved sampling across four windows (during exercise, immediately post-exercise, 24 h, 72 h) resolves temporal misalignment; (iii) causal re-stratification of legacy individual-participant data via target trial emulation and causal mediation enables retrospective de-confounding of existing meta-analytic evidence. %VO_2_max, percentage of maximal oxygen uptake; %1RM, percentage of one-repetition maximum; HIIT, high-intensity interval training; DAMPs, damage-associated molecular patterns; M1/M2, pro-inflammatory/repair macrophage phenotypes; IL-6, interleukin-6; AMPK, AMP-activated protein kinase; IL-10, interleukin-10; IL-1ra, interleukin-1 receptor antagonist.

We propose that a substantial proportion of the extreme heterogeneity observed in exercise–cytokine meta-analyses may arise from the conflation of metabolic and mechanical axes into a single exposure category, while also acknowledging contributions from other sources such as participant characteristics, intervention dose, and assay methods. Accordingly, this article focuses on the mechanistic divergence between these two axes and discusses its implications for interpreting exercise-induced inflammatory outcomes (**Figure 1**).

## 2 Mechanistic divergence

### 2.1 Metabolic axis

When aerobic exercise is sustained at moderate metabolic intensity (≥30 min, 40–60% VO_2_max), continuously contracting skeletal muscle fibers release IL-6 into the circulation, with output proportional to exercise duration and the mass of recruited muscle. Importantly, this IL-6 differs fundamentally from that observed in chronic inflammation (12): myokine-derived IL-6 primarily signals via the membrane-bound IL-6 receptor (mIL-6Rα), activating the classic signaling pathway and downstream gp130/JAK–STAT3 cascade, rather than the soluble receptor–mediated trans-signaling pathway characteristic of pathological inflammation (2, 12).

Accordingly, myokine IL-6 can be conceptualized as a transient energy-regulatory factor, facilitating substrate redistribution during physical activity while temporarily suppressing immune activation (4). Downstream, it activates AMP-activated protein kinase (AMPK) and upregulates anti-inflammatory cytokines—primarily IL-10 and the IL-1 receptor antagonist (IL-1ra)—which in turn inhibit TNF-α production by monocytes and macrophages (12).

This pathway differs from resistance exercise along three key dimensions. First, at the level of metabolic gating, IL-6 release is modulated by glycogen availability; under low-glycogen conditions, IL-6 output can increase several-fold (13), indicating that the anti-inflammatory signal is driven by substrate availability rather than mechanical load. Second, its effects are systemic rather than localized: once IL-10 and IL-1ra enter the circulation, they act on resident macrophages in distal tissues such as adipose tissue and liver, suppressing TNF-α production and thereby alleviating chronic low-grade systemic inflammation associated with metabolic syndrome (12). Third, the temporal profile is rapid and transient: circulating IL-6 peaks during exercise and returns to baseline within 1–2 hours post-exercise, followed by a brief elevation of IL-10 and IL-1ra that quickly resolves (14). This indicates that the entire anti-inflammatory cascade is largely completed before the inflammatory response of the mechanical axis is initiated.

### 2.2 Mechanical axis

Resistance exercise performed at moderate-to-high mechanical loads (60–80% 1RM), particularly when dominated by eccentric contractions, induces microstructural muscle damage, including sarcolemmal disruption, Z-line streaming, and extracellular matrix perturbation (15). This damage triggers the release of endogenous danger signals—damage-associated molecular patterns (DAMPs)—which activate resident immune cells via pattern recognition receptors and initiate neutrophil infiltration within 2–6 hours post-exercise (16).

Subsequently, pro-inflammatory M1 macrophages accumulate, peaking at 24–48 hours, and mediate phagocytic clearance of damaged tissue (15). Under the influence of local IL-10, IL-13, and growth factor signaling, macrophages then transition from the M1 to the M2 phenotype, shifting the tissue microenvironment from inflammatory clearance to regenerative repair (15).

Thus, the immunological profile of this pathway is inherently biphasic: an initial pro-inflammatory phase—characterized by elevations in IL-1β, TNF-α, and locally produced IL-6—persists for 24–72 hours, followed by a resolution and remodeling phase (17), as confirmed by meta-analyses of acute resistance exercise (11). While this biphasic response is adaptive and underlies muscle hypertrophy and connective tissue remodeling, its direction at any single acute time point is often opposite to that of the aerobic anti-inflammatory cascade.

Moreover, IL-6 generated in this context originates from infiltrating leukocytes and damaged muscle fibers rather than intact contracting fibers, and its signaling milieu is distinct: it is co-expressed with TNF-α and IL-1β in an NF-κB–dependent inflammatory environment, driving canonical pro-inflammatory signaling rather than AMPK-mediated anti-inflammatory responses (4). A computational analysis integrating 75 transcriptomic datasets demonstrated that acute exercise induces an immediate surge in M1 macrophage signatures, whereas chronic training promotes sustained M2 activation (18). Correspondingly, repeated resistance training attenuates the acute inflammatory response through the “repeated bout effect” (17); however, these chronic adaptations have no direct analogue within the metabolic axis.

### 2.3 Temporal misalignment as a source of measurement artefact

The temporal divergence between the metabolic and mechanical axes introduces measurement artefacts when inflammatory outcomes are pooled. For example, if IL-6 is assessed 24 hours post-exercise, the myokine-derived IL-6 surge from aerobic exercise has already resolved, and the measured value approximates baseline. In contrast, resistance exercise remains within the peak phase of damage-induced inflammation, capturing tissue-derived, NF-κB–mediated pro-inflammatory IL-6 (17). Pooling these signals under a single category of “moderate-intensity exercise–induced IL-6 response” inevitably conflates temporally and mechanistically distinct processes, inflating variance.

This issue becomes even more complex in chronic intervention trials. If the final blood sampling occurs within 72 hours of the last training session, the resistance training group may still exhibit residual acute-phase inflammation, rather than reflecting true chronic adaptation (19, 20).

### 2.4 From pathway divergence to meta-analytic heterogeneity

Integrating the metabolic and mechanical pathways and their distinct temporal dynamics provides a structural explanation for the heterogeneity patterns observed in the literature. Notably, the heterogeneity of CRP in meta-analyses (I² = 72%) is substantially lower than that of IL-6 (I² = 97%) and TNF-α (I² = 91%). CRP, as a hepatic acute-phase protein regulated by circulating IL-6 through a relatively slow feedback loop (half-life ~19 hours) (21), integrates the anti-inflammatory contributions of both metabolic and mechanical axes over repeated exercise sessions spanning days to weeks. Consequently, it is less sensitive to modality-specific differences in single bouts, and meta-analyses tend to report relatively consistent reductions.

In contrast, IL-6 and TNF-α occupy signaling positions that are highly modality-sensitive: IL-6 functions as an anti-inflammatory myokine along the metabolic axis but as a local damage signal along the mechanical axis; TNF-α is suppressed along the metabolic axis yet transiently elevated along the mechanical axis (11, 14). When studies from these two modalities are pooled without stratification, opposing effects may partially cancel each other, leading to inflated I² values and wider confidence intervals. This reflects not only methodological limitations, but also the inappropriate aggregation of orthogonal biological processes.

However, restricting meta-analyses to a single exercise modality is insufficient to eliminate heterogeneity. In the aerobic-only meta-analysis by Zheng et al. (2019), heterogeneity remained extremely high, with I² values of 99%, 97%, and 98% for CRP, IL-6, and TNF-α, respectively (22). Similarly, in the resistance training–only meta-analysis by Kim and Yeun (2022), heterogeneity persisted, with I² values of 84% for IL-6, 81% for TNF-α, and a moderate 69% for CRP (23). These findings indicate that the conflation of aerobic and resistance exercise is neither the sole nor the dominant source of heterogeneity. Additional contributors include within-modality variability in intervention protocols—for example, Zheng et al. pooled heterogeneous “aerobic” interventions such as Tai Chi, treadmill walking/jogging, step exercise, and cycle ergometry (22)—as well as differences in participant characteristics (age, comorbidities, baseline inflammatory status), assay platforms and analytical methods, and the timing of blood sampling relative to the last exercise session. Notably, temporal misalignment has been explicitly identified as a contributor to between-study variability (22).

These observations suggest that even within a single modality, post-exercise inflammatory responses should be interpreted along a temporal trajectory rather than collapsed into a single outcome label. Importantly, axis-level misclassification and temporal misalignment are not competing explanations, but rather two interrelated aspects of the same problem.

## 3 Discussion

We recommend that the term “moderate-intensity exercise” should not be used as a standalone pooled exposure category in evidence synthesis (e.g., meta-analytic search and aggregation strategies) without prior axis-based stratification. We do not advocate removing this term from the exercise science lexicon; rather, when it is used as a criterion for pooling in meta-analyses or systematic reviews, primary studies should first be stratified along metabolic and mechanical axes before aggregation, to avoid treating physiologically incommensurable stimuli as equivalent exposures.

Accordingly, exercise interventions should be reported with clear differentiation between metabolic load metrics (%VO_2_max, METs, exercise duration) and mechanical load metrics (%1RM, volume load, eccentric component). This can build upon existing reporting frameworks such as the Consensus on Exercise Reporting Template (CERT) (24), with the addition of explicit requirements for independent reporting and evaluation of metabolic and mechanical axes.

The metabolic and mechanical axes should not be viewed as mutually exclusive categories, but rather as orthogonal dimensions within a two-dimensional coordinate system, in which any exercise bout can be independently quantified along both axes. Thus, studies dominated by aerobic modalities (e.g., running, cycling, brisk walking) should primarily characterize the metabolic axis using %VO_2_max, %HRmax, or METs, alongside duration (7). In contrast, resistance-dominant interventions, particularly those with substantial eccentric components, should primarily characterize the mechanical axis using %1RM (7), volume load (25), and eccentric proportion (26). For mixed-modality interventions that impose substantial loads on both axes—such as high-intensity interval training (HIIT) (27), circuit resistance training (28, 29), plyometrics (30), and downhill running (31)—both dimensions should be reported concurrently rather than forced into a single-axis classification.

In evidence synthesis, such interventions should either be treated as a distinct stratum or incorporated into meta-regression models with both axes represented as continuous effect modifiers (32, 33), thereby reducing mechanism-driven heterogeneity.

Additionally, study designs should explicitly account for the temporal sequencing of metabolic and mechanical responses to avoid overlap. A single time point is insufficient to capture the full cytokine trajectory (34, 35). We therefore recommend that randomized controlled trials assessing inflammatory outcomes include at least four sampling windows: during exercise (capturing the myokine surge), immediately post-exercise, 24 hours post-exercise (capturing the peak of damage-induced responses in resistance modalities), and 72 hours post-exercise (capturing the resolution/M2 phase). This approach minimizes signal overlap and improves interpretability.

Furthermore, resolving heterogeneity in existing meta-analyses cannot rely solely on generating new primary studies; re-analysis of existing data is also required. We propose applying target trial emulation frameworks (36) and causal mediation analysis (37) to individual participant data meta-analyses, modeling exercise modality as an effect modifier rather than collapsing it into a single exposure. This approach may help disentangle the interacting contributions of metabolic and mechanical axes and reduce heterogeneity arising from their conflation.

## 4 Conclusion

The anti-inflammatory effects of exercise are well established; however, the classification frameworks underlying current evidence synthesis contain critical limitations. While “moderate-intensity exercise” remains a useful operational term in clinical communication, its use as a pooling criterion in evidence synthesis conflates metabolically and mechanically distinct, orthogonal stimuli into a single exposure category. These stimuli can elicit divergent—and even opposing—acute cytokine responses. Consequently, the observed heterogeneity in cytokine outcomes reflects not only statistical noise but also misclassification at the level of evidence integration. Therefore, the key to interpreting exercise-induced immune responses lies not in increasing data volume, but in identifying and disentangling the distinct biological processes that underlie them, thereby minimizing mechanism-driven heterogeneity.

## Data Availability

The original contributions presented in the study are included in the article/supplementary material. Further inquiries can be directed to the corresponding authors.
